# Detecting Susceptibility to Breast Cancer with SNP-SNP Interaction Using BPSOHS and Emotional Neural Networks

**DOI:** 10.1155/2016/5164347

**Published:** 2016-05-11

**Authors:** Xiao Wang, Qinke Peng, Yue Fan

**Affiliations:** Systems Engineering Institute and School of Electronic and Information Engineering, Xi'an Jiaotong University, Xi'an, Shaanxi 710049, China

## Abstract

Studies for the association between diseases and informative single nucleotide polymorphisms (SNPs) have received great attention. However, most of them just use the whole set of useful SNPs and fail to consider the SNP-SNP interactions, while these interactions have already been proven in biology experiments. In this paper, we use a binary particle swarm optimization with hierarchical structure (BPSOHS) algorithm to improve the effective of PSO for the identification of the SNP-SNP interactions. Furthermore, in order to use these SNP interactions in the susceptibility analysis, we propose an emotional neural network (ENN) to treat SNP interactions as emotional tendency. Different from the normal architecture, just as the emotional brain, this architecture provides a specific path to treat the emotional value, by which the SNP interactions can be considered more quickly and directly. The ENN helps us use the prior knowledge about the SNP interactions and other influence factors together. Finally, the experimental results prove that the proposed BPSOHS_ENN algorithm can detect the informative SNP-SNP interaction and predict the breast cancer risk with a much higher accuracy than existing methods.

## 1. Introduction

Breast cancer is a major cause of death among women. Some genes (e.g., BRCA1 and BRCA2) have already been known as the cause of breast cancer [1]. However, only 5% case of breast cancer has these mutations; in this way, these symbols failed to be used for most of women. Fortunately, besides, on these rare mutations, increasing evidence shows that the risk of breast cancer can be measured by the SNPs, which is one of the most common types of mutations for human being [2–4]. Furthermore, with the development of SNP microarray and genome-wide associations studies (GWAS), the research about breast cancer and SNPs becomes more popular.

For complex disease like breast cancer, the effect of an individual SNP is small. Researchers generally focus on the joint genetic effect of SNP combinations which may increase susceptibility to the cancer. However, most of studies just care about the whole set of disease related SNPs and treat all the SNPs in the dataset equally [5]. In this way, they fail to consider the small scale interaction among SNPs. On the other hand, increasing evidence proves that SNP-SNP interactions exist [6–8]. To change this situation, a method to use the SNP-SNP interaction in breast cancer susceptibility identification is in need.

With the development of microarray, one of the most important challenges to detect SNP-SNP interactions is the complex combination of data with increasing SNPs number. In order to improve the effectiveness to identify these interactions, researchers try to use many different algorithms, for example, [9] uses the Genetic Algorithm (GA), [10] uses the Particle Swarm Optimization (PSO) algorithm, and [11] uses the Polymorphism Interaction Analysis (PIA). These methods have the ability to detect SNP interactions in high dimensional dataset; however, due to the random generator initial values and optimizing process, they generally need lots of iterations and are easily trapped into the local optima. So an improved algorithm for solving this puzzle is essential. Here, we propose BPSOHS to improve the performance of identifying the SNP interactions. Inspired by the decision-making process in the real society, we divide the particle swarm as “leader” part and “follower” part, which have different evolutionary strategies. Our former work proved that this novel method is faster than other swarm intelligent algorithms and much easier to converge to the globally optimal solution [12]. So, by this improved PSO, after encoding and matching, we can improve the performance of identifying the SNP interactions related to the cancer.

The defined SNP-SNP interaction is an important tendency factor about the breast cancer [13]. However, there is still no specific method to use this kind of information into the cancer susceptibility. ENN is a novel method inspired by the emotional process of the human brain, which is usually used in the face recognition [14, 15]. In this paper, by designing the emotional value based on the SNP interactions, we explore the range of ENN and use it for susceptibility analysis. So, in this novel ENN, the SNP interaction features can be regarded as the prior tendency of classification and influence the result of classification, just like what emotions do in the process of people making choices.

In the end, we propose a pipeline for the selection and usage of SNP-SNP interactions. At the beginning, the BPSOHS algorithm is used to identify the informative SNP combinations related to the cancer. Then, we transform these new features as an additional vector of each sample. Finally, by treating the vector as tendency emotional value in the ENN, the novel network can particularly consider the SNP-SNP interaction in a more directly and quickly way. According to the output of the networks, our method can measure the breast cancer risk for each sample. The case-control study in 10000 people suggests that our pipeline can detect the useful SNP interactions and effectively consider them in susceptibility analysis to breast cancer.

## 2. Material and Methods

### 2.1. Dataset Preparation

The datasets we use in this paper were obtained from the breast cancer case-control study in [16]. This dataset has 5000 controls and 5000 cases, with the SNPs selected by the biological research.

In order to be convenient for the further discussion, we assume that the dataset has *m* samples and each sample has *n* SNPs. The SNP value can be represented as ∑ = {1,2, 3}, where 1 represents the major homozygous sites, 2 represents the minor homozygous sites, and 3 represents the heterozygous sites. Then, *S*_*i*_ = (*s*_*i*1_, *s*_*i*2_,…, *s*_*in*_) denote an individual sample and *D* = (*S*_1_, *S*_2_,…, *S*_*m*_) represent the whole dataset.

### 2.2. Particle Swarm Optimization

PSO is a swarm algorithm developed by Kennedy and Eberhart [17]. For basic PSO, each solution is corresponding to the position of a particle, and the velocity and direction of each particle can be adjusted according to both its own parameter and the best particles in the swarm. The idea of this process is particles “flying” through a multidimensional possible search space and looking for the global optimum. By both individual memory and global memory, a particle calculates and moves to its next position. The basic elements of PSO are described as follows:

(1) vijt+1=vijt+c1r1pijt−xijt+c2r2pgjt−xijt,xijt+1=xijt+vijt+1,

where *v*_*ij*_ and *x*_*ij*_ are the velocity and position of the *i*th particle in *j*th bit, *r*_1_, *r*_2_ are the random numbers within [0,1], and *c*_1_, *c*_2_ are the acceleration constants. *p*_*ij*_ is the best position of the *i*th particle in *j*th bit, and *p*_*gj*_ is the *j*th bit of best position of the best particle in the swarm. The velocity *v*_*ij*_ is within [−*V*_max_, *V*_max_] to make sure that the particle is flying in the range of possible solution space.

### 2.3. Binary Particle Swarm Optimization with Hierarchical Structure

The basic PSO is just designed for the continuous problems. In order to make it more widely used, in 1997, Kennedy and Eberhart propose BPSO (binary particle swarm optimization) to use in the discrete problems [18]. Different from the basic conventional PSO, in BPSO, each bit of a particle can just move in a state space to 0 or 1 as in the following function:

(2) $$\begin{array}{c} x_{ij}=\left\{\begin{array}{cc} 1 & if\;\;rand\left(\right)<S\left(v_{ij}\right),\\ 0 & else. \end{array}\right. \end{array}$$

In this function, *S*(*v*_*ij*_) = 1/(1 + *e* ^−*v*_*ij*_^) denote the probability of *x*_*ij*_ choosing 1 and rand() is a random real number within [0,1].

In basic PSO and BPSO algorithm, the particles are treated equally. However, this process works much differently from the real society. The researchers about sociology point out that the leaders in the group seem to have the stronger say in making decision. Most of people prefer to follow their leaders, so the particles seem to also have status in the swarm [19]. Inspired by this idea, we propose BPSOHS [12] with two kinds of particles: “leader” particles and “follower” particles, and they can be regarded as the “leader” and “followers” in the society.

At the beginning, there are *K* particles defined as “leader” in the swarm, while others are “follower.” According to the fitness function, the “leader” and “follower” can switch in these two statuses, to make sure that the better particles are “leaders.” Thus, at the *t*th iteration, the followers can walk toward the leaders based on the following formula:

(3) probijFt+1=min⁡probijFt+1αLIF,1,if  ∑j=1KxijLtK≥0.5,max⁡0,probijFt−1αLIF,if  ∑j=1KxijLtK<0.5,vijFt+1=−Vmax,if  probijFt+1=0,S−1probijFt+1,else,Vmax,if  probijFt+1=1,xijFt+1=1,rand<probijFt+1,0,else,

where *S* ^−1^(*x*) = ln(*x*/(1 − *x*)), *L* indicates the “leader,” and *F* indicates the “follower.” And *α*_LIF_ is a parameter to limit the followers' speed. According to these formulas, when we update the position of particle, we need consider both its own position and the decisions of “leader.”

### 2.4. Encoding Schemes and Performance Measurement

In BPSOHS, the *d*th SNPs in *i*th particle can be represented by two bits: *X*_*id*_ = (*S*_*d*1_, *S*_*d*2_)_*id*_. *S*_*d*1_ and *S*_*d*2_ can be 0 and 1, so a SNP can be represented as follows:

(4) Sd1,Sd2id=0,0,non-selected  dth  SNP,0,1,selected  dth  SNP  and  Genotype  as  1,1,0,selected  dth  SNP  and  Genotype  as  2,1,1,selected  dth  SNP  and  Genotype  as  3.

In this way, the combination of (*S*_*d*1_, *S*_*d*2_) can represent the four different states of the SNPs. The *i*th particle can be described by

(5) $$\begin{array}{c} X_{i}=\left\{\;\left(\;S_{11},S_{12}\;\right),\left(\;S_{21},S_{22}\;\right),\ldots ,\left(\;S_{n1},S_{n2}\;\right)\;\right\}. \end{array}$$

For example, if *X*_*i*_ = {(0,0), (1,1), (0,0), (0,1)}, this particle represents that we choose the 2nd and 4th SNP in genotypes 3 and 1. Thus, the phenotype of samples can be described by the particles. In this BPSOHS process, the value of fitness function presents the importance of particle. In the susceptibility analysis, we employ the SNP-SNP interactions to influence the tendency of the classification; thus, the ratio difference between the cases and controls is useful. Referring to [9], we use the following formula to measure the importance of SNP interactions:

(6) $$\begin{array}{c} F\left(\;C_{i}\;\right)=\frac{n\left(case\cap C_{i}\right)}{n\left(case\right)}-\frac{n\left(control\cap C_{i}\right)}{n\left(control\right)}, \end{array}$$

where *n* denote the elements number in a dataset, while case ∩*C*_*i*_ is the dataset subset in the breast cancer case group with specific interaction *C*_*i*_, and control∩*C*_*i*_ is the dataset subset in the control group with specific interaction *C*_*i*_.

By using this fitness function and BPSOHS, we get some SNP-SNP interactions in significant association with breast cancer; the details of some of them are shown in Table 1.

In addition to these interactions in 2 or 3 interactions, we still find some interactions with more SNPs. For example, the combination, rs3020314 (CT)-rs1543404 (TC)-rs2747652 (CT)-rs9340799 (AG)-rs1709182 (TT)-rs9478249 (TG)-rs660149 (CC)-rs11571171 (TT)-rs858518 (TC)-rs858524 (AG)-rs2017591 (TT), appears 7 times in the case group and only once in the control group.

### 2.5. Emotional Neural Networks Based on SNP-SNP Interactions

**Figure 1:**
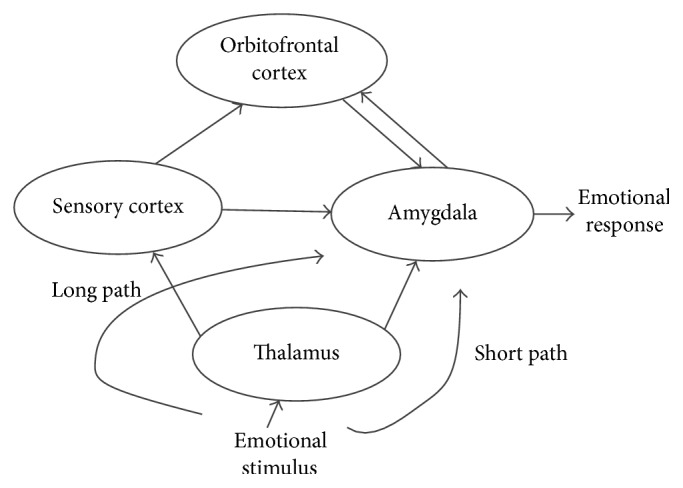
Simple modeling of emotional brain.

Neural networks are popular in the field of bioinformatics and disease susceptibility analysis. However, the basic fully connected feed forward neural networks just treat all the input in the same level. Although the powers will adjust by the importance of input data, this architecture still fails to use the inside information about data. Emotional neural networks are a novel method to deal with this problem. According to the related works [20], researches about brain characterize that there are a short path for the emotional signal in brain, which help the feedback become more directly and quickly. The simple modeling of the emotional brain is shown in Figure 1.

**Figure 2:**
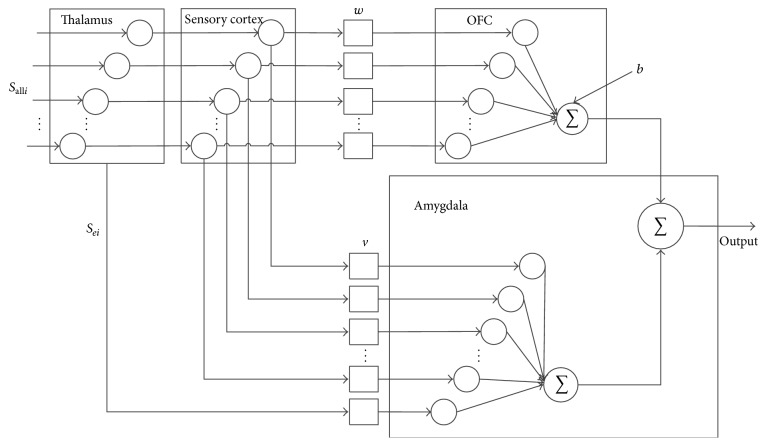
Emotional neural networks for susceptibility analysis to breast cancer.

Referring to the works of Lotfi and Akbarzadeh-T [14], we can use the neural network in Figure 2 to simulate this architecture. In the long path from the thalamus, sensory cortex OFC to the amygdala is the path for the general input. And the short path from the thalamus directly to the amygdala is the path for the tendency emotional input.

In the related works about ENN architecture [21, 22], the input of the sensory cortex and amygdala is the same, which is different from the real process of our brain. Generally, in the human emotional brain, the emotional value is the a priori tendency information [23, 24]. So, in this paper, based on the BPSOHS and the SNP-SNP interaction detection, we try to use this kind of a priori information in susceptibility analysis. As it is shown in Table 1, the specific phenotype has different emergence probability in case and control class. So these SNP-SNP interactions can influence the cancer risk of the samples.

Assume that we use *p* SNP-SNP interactions as the emotional value; then, the tendency vector is as follows: *S*_*ei*_ = (*s*_*ei*1_, *s*_*ei*2_,…, *s*_*eiq*_,…, *s*_*eip*_). According to (7), we can get the final sequence, *S*_all*i*_ = (*S*_*i*_, *S*_*ei*_) = (*s*_*i*1_, *s*_*i*2_,…, *s*_*in*_, *s*_*ei*1_,…, *s*_*eiq*_,…, *s*_*eip*_), as follows:

(7) $$\begin{array}{c} s_{eiq}=\left\{\begin{array}{cc} s_{oeq}, & is\;\;the\;\;specific\;\;phenotype\\ 0, & otherwise, \end{array}\right. \end{array}$$

where *s*_*oeq*_ is the odds ratio of the *q*th interaction, which can measure its importance. Inspired by the ENN architecture focus on the image recognition [25], we design the output of the thalamus as *S*_*ei*_ = *s*_*ei*1_,…, *s*_*eiq*_,…, *s*_*eip*_, and the final output of the neural networks is calculated by

(8) E1=hardlim⁡∑i=1n+pvi×Si+∑i=n+p+1n+2pvi×Sei−hardlim⁡∑i=1nwi×Si,

where *v*_*i*_ is the weight for the amygdala, while *v*_*i*_, *i*⊆[1, *n* + *p*], is the related weight of *S*_all_, and *v*_*i*_, *i*⊆[*n* + *p* + 1, *n* + 2*p*], is the related weight about the emotional value *S*_*ei*_. *w*_*i*_ is the weight for the OFC.

In this architecture, these parameters are updated as in the following formula:

(9) vi=1−γvi+αmax⁡R1−Ea1,0Sifor  i=1,…,n+p,vi=1−γvi+αmax⁡R1−Ea1,0Sei−n−pfor  i=n+p+1,…,n+2p,wi=1−γwi+βR0Sifor  i=1,…,n+p,

where the learning rates are *α* and *β*. The decay rate is *γ*. *R*_1_ is a binary input about reinforce, and *R*_0_ is the reward value calculated according to the BELPIC model [26]:

(10) $$\begin{array}{c} R_{0}=\left\{\begin{array}{cc} \max\left(Ea_{1}^{\prime }-R_{1},0\right)-Eo_{1} & if\;\;\left(R_{1}=1\right)\\ \max\left(Ea_{1}^{\prime }-Eo_{1}\right) & if\;\;\left(R_{1}=0\right), \end{array}\right. \end{array}$$

where

(11) Ea1′=hardlim⁡∑i=1n+pvi×Si,Eo1=hardlim⁡∑i=1n+pwi×Si.

In the training process, the value *R*_1_ = 1 is used when the goal is presented, while the value *R*_1_ = 0 is used for other situations. In the test process, the final output *E* is calculated according to (8). By this process, we treat the SNP interactions as the emotional value and use these tendency features to reflect the final output of the neural network. By this architecture, we get the score to measure the risk for breast cancer, and the details will be shown in the next section.

## 3. Results and Discussion

### 3.1. Evaluation Function

To measure the performance of the SNP-SNP interactions and the novel neural networks, we need an effective evaluation function. Referring to other works about classification, in this paper, we use Sn (sensitivity), Sp (specificity), Acc (accuracy), RR (risk ratio), and OR (odds ratio); they are defined as follows:

(12) Sensitivity=TPTP+FN,Specificity=TNFP+TN,Accuracy=TP+TNTP+FN+FP+TN,Risk  Ratio=TP×FP+TNFP×TP+FN,Odds  Ratio=TP×TNFP×FN,

where TP (True Positive) is the number of positive samples that are predicted as positive, TN (True Negative) is the number of negative samples that are predicted as negative, FP (False Positive) is the number of negative samples that are predicted as positive, and FN (False Negative) is the number of positive samples that are predicted as negative.

### 3.2. Experimental Results and Comparison

In order to prove that the SNP-SNP interaction feature is useful for the susceptibility analysis, we use 5-fold cross-validation to evaluate the performance of our method. We compare the performance of some popular basic neural networks (BP (Back Propagation), RBF (Radial Basis Function), and PNN (Pattern Recognition Neural networks)) with or without the novel information. The details of the experiment are shown in Table 2.

**Figure 3:**
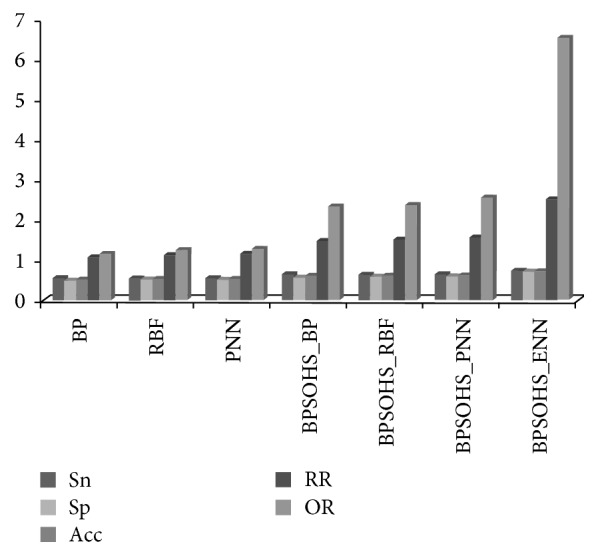
Bar graph of the performance of different methods.

Figure 3 shows that these methods use the SNP-SNP interaction features to achieve better performance than those methods which do not use the small scale information. By considering this tendency information in the specific architecture, our BPSOHS_ENN gets the best performance compared with these basic methods.

To prove that our method is suitable for this problem, we also use our method to compare with some newly published papers. Majority of these researches just give useful SNP interaction, while only part of samples has these interactions. So, to make sure of the fairness of the comparison, we filter these results based on interactions that only appeared in less than 10% of samples, and in Table 3 we compare our result with the best of the remaining ones.

In Table 3, IBBA method uses the dataset derived by 7 SNPs from CXCL12-related genes, and IGA and IPSO use the same dataset as this paper. The tests on the different dataset also prove the effectiveness of our method. By using the small scale feature and other features in the same neural network architecture, the process of the susceptibility analysis becomes much easier. The tendency of SNP combination can give a more reasonable initial value for the classifier. So consider this a priori information in a fast and direct way to help improve the performance of the neural network.

The experimental results prove that our method has the power to consider these useful SNP-SNP interactions together, which is much better than these single barcodes. What is more, Table 3 shows that our method is competitive compared with the other published methods. Compared with them, our method has significant advantages in identifying the case and control, which is useful for us to predict and prevent the cancer.

## 4. Conclusions

The susceptibility analysis to disease is normally based on a group of SNPs. However, the small scale relationship in the group is rarely being mentioned. In this paper, we use the BPSOHS to pick out the neglected small scale SNP-SNPs. In order to consider the relationship about the SNP interaction, we propose specific partially connected architecture neural networks. By simulating the process of human brain deal with the emotional value, the related SNPs have the chance to work together with other normal features to calculate possibility of the samples suffering from breast cancer. According to the cancer-related SNP interactions and output of the novel neural network, we can measure the cancer risk of the samples, which is useful for us to prevent the possible cancer.

## Figures and Tables

**Table 1:** SNP interactions about breast cancer.

SNP (gene) (chromosome/position)	SNP type	Case number	Control number	Difference	Odds ratio	95% CI	*P* value
rs3020314 (ESR1) (6/152270672) rs500760 (PGR) (11/100909991)	CC- AA	1230	1404	174	0.836	0.7644–0.9135	0.001

rs3020314 (ESR1) (6/152270672) rs2017591 (STS) (X/7158114)	CC- TC	984	1152	168	0.818	0.7436–0.9008	0.003

rs3020314 (ESR1) (6/152270672) rs2077647 (ESR1) (6/152129077)	CT- GG	1055	1213	158	0.835	0.7602–0.9170	0.008

rs500760 (PGR) (11/100909991) rs2017591 (STS) (X/7158114)	AG- CC	1326	1476	150	0.862	0.7896–0.9404	0.001

rs2077647 (ESR1) (6/152129077) rs2017591 (STS) (X/7158114)	AG- TC	1117	1263	146	0.851	0.7762–0.9333	0.040

rs3020314 (ESR1) (6/152270672) rs660149 (PGR) (11/100934314) rs11571171 (PGR) (11/100974887)	CT- CC- TT	602	509	93	1.208	1.0657–1.3687	0.003

rs3020314 (ESR1) (6/152270672) rs500760 (PGR) (11/100909991) rs2017591 (STS) (X/7158114)	CC- AA- TC	571	699	128	0.793	0.7048–0.8929	0.012

rs6269 (COMT) (22/19949952) rs2175898 (ESR1) (6/152196952) rs660149 (PGR) (11/100934314)	AA- AG- CC	1035	1158	123	0.866	0.7877–0.9522	0.003

rs3020314 (ESR1) (6/152270672) rs1543404 (ESR1) (6/152428838) rs2747652 (ESR1) (6/152437016) rs9340799 (ESR1) (6/152163381) rs1709182 (ESR1) (6/152175357) rs9478249 (ESR1) (6/152194431) rs660149 (PGR) (11/100934314) rs11571171 (PGR) (11/100974887) rs858518 (SHBG) (17/7533025) rs858524 (SHBG) (17/7511287) rs2017591 (STS) (X/7158114)	CT- TC- CT- AG- TT- TG- CC- TT- TC- AG- TT	7	1	6	7.01	0.8800–151.68	0.034

**Table 2:** The performance of ENN compared with basic neural networks.

Method	Sn	Sp	Acc	RR	OR
BP	0.5486	0.4864	0.5175	1.0681	1.1505
RBF	0.5394	0.5152	0.5273	1.1126	1.2445
PNN	0.5448	0.5066	0.5257	1.1508	1.2808
BPSOHS_BP	0.6462	0.5606	0.6034	1.4706	2.3302
BPSOHS_RBF	0.6290	0.5826	0.6058	1.5069	2.3664
BPSOHS_PNN	0.6406	0.5888	0.6147	1.5579	2.5523
BPSOHS_ENN	0.7268	0.7106	0.7187	2.5114	6.5322

**Table 3:** The performance of ENN compared with published methods.

Method	Sn	Sp	Acc	RR	OR
IGA [9]	—	—	0.5351	1.08	1.17
IPSO [16]	—	—	0.49	0.82	0.79
IBBA [27]	0.132	0.940	0.619	—	2.384
BPSOHS_ENN	0.7268	0.7106	0.7187	2.5114	6.5322
